# NLPR3 inflammasome inhibition alleviates hypoxic endothelial cell death in vitro and protects blood–brain barrier integrity in murine stroke

**DOI:** 10.1038/s41419-021-04379-z

**Published:** 2021-12-20

**Authors:** Maximilian Bellut, Lena Papp, Michael Bieber, Peter Kraft, Guido Stoll, Michael K. Schuhmann

**Affiliations:** 1 Department of Neurology, University Hospital Wuerzburg, Josef-Schneider-Str. 11, 97080 Würzburg, Germany; 2 Department of Neurology, Klinikum Main-Spessart, Grafen-von-Rieneck-Str. 5, 97816 Lohr, Germany

**Keywords:** Stroke, Preclinical research, Inflammasome

## Abstract

In ischemic stroke (IS) impairment of the blood–brain barrier (BBB) has an important role in the secondary deterioration of neurological function. BBB disruption is associated with ischemia-induced inflammation, brain edema formation, and hemorrhagic infarct transformation, but the underlying mechanisms are incompletely understood. Dysfunction of endothelial cells (EC) may play a central role in this process. Although neuronal NLR-family pyrin domain-containing protein 3 (NLRP3) inflammasome upregulation is an established trigger of inflammation in IS, the contribution of its expression in EC is unclear. We here used brain EC, exposed them to oxygen and glucose deprivation (OGD) in vitro, and analyzed their survival depending on inflammasome inhibition with the NLRP3-specific drug MCC950. During OGD, EC death could significantly be reduced when targeting NLRP3, concomitant with diminished endothelial NLRP3 expression. Furthermore, MCC950 led to reduced levels of Caspase 1 (p20) and activated Gasdermin D as markers for pyroptosis. Moreover, inflammasome inhibition reduced the secretion of pro-inflammatory chemokines, cytokines, and matrix metalloproteinase-9 (MMP9) in EC. In a translational approach, IS was induced in C57Bl/6 mice by 60 mins transient middle cerebral artery occlusion and 23 hours of reperfusion. Stroke volume, functional outcome, the BBB integrity, and—in good agreement with the in vitro results—MMP9 secretion as well as EC survival improved significantly in MCC950-treated mice. In conclusion, our results establish the NLRP3 inflammasome as a critical pathogenic effector of stroke-induced BBB disruption by activating inflammatory signaling cascades and pyroptosis in brain EC.

## Introduction

Endothelial cells (EC) play a central role in maintaining blood–brain barrier (BBB) integrity. In ischemic stroke (IS), which is a leading cause of death and disability worldwide, the BBB is progressively impaired and its breakdown is associated with poor long-term outcomes and adverse treatment effects such as intracranial hemorrhages [1, 2]. There is increasing evidence that inflammatory processes contribute to stroke development and bleeding complications [3–7], but the role of EC within these has been largely neglected.

Inflammasomes in general, and the NLR-family pyrin domain-containing protein 3 (NLRP3) inflammasome in particular, are molecular protein complexes that sense cellular deviation from homeostasis as a danger signal and subsequently initiate inflammatory responses [8]. They are responsible for the activation of Caspase 1 and its downstream pro-inflammatory cytokines interleukin (IL) 1b, IL18, and the pyroptosis-inducing Gasdermin D (GSDMD). Recently, we have shown the potential of NLRP3 inhibition to reduce infarct development during IS [9]. NLRP3 was expressed in neurons and EC. NLRP3-induced inflammation plays a detrimental role in systemic endothelial dysfunction (e.g., large vessel disease, arteriosclerosis) [10], but the role of endothelial NLRP3 induction in stroke and its impact on BBB integrity is largely unknown.

Here, we show that the inhibition of NLRP3 prevents hypoxic/ischemic BBB disruption by reducing EC death mediated through the pyroptosis pathway.

## Materials and methods

### Materials

All used materials are separately listed in detail in the Supplement.

### Animals

Animal experiments were approved by local governmental authorities (Regierung von Unterfranken) and conducted in accordance with the US National Institutes of Health Guidelines for the Care and Use of Laboratory Animals. Moreover, the experiments were designed, performed, and reported according to the Animal Research: Reporting of In Vivo Experiments guidelines [11]. We used 6–8-week old male C57Bl/6N mice, purchased from Charles River Laboratories (Sulzfeld, Germany). In all, 10 mice were used for infarct size measurement, clinical tests, and immunohistochemistry (five treated with vehicle, five with MCC950). 22 animals (11 treated with MCC950, 11 treated with vehicle) were used for matrix metalloproteinase-9 (MMP9) measurement. Out of these 22 animals, 14 (seven treated with MCC950, seven treated with vehicle) were used for Western Blot analysis. After randomization,transient middle cerebral artery occlusion (tMCAO) was conducted for 60 min. Surgery and evaluation of all readout parameters were performed blinded to the experimental groups. The group sizes were derived from previous tMCAO-studies, which showed significant effects with a power of 0.8 and a probability of a type I error of <0.5 [9, 12].

### Animal treatment

Animals were treated with 100 µl of the inflammasome-inhibitor MCC950 or the same volume of vehicle (1× PBS), which were administered by an intraperitoneal injection directly before occluding the MCA for 60 min [9].

### Ischemia model

The tMCAO model was used to induce focal cerebral ischemia as described before [13]. The experiments were carried out blinded. An independent researcher not involved in data analysis coded and randomized the mice. To reduce the variability of our outcome parameters caused by sex differences only male mice were used [14]. Before tMCAO, the mice were anesthetized with 2.0% isoflurane in O_2_ (v/v). 200 mg/kg body weight metamizol was injected subcutaneously and 4% lidocaine gel was applied on the wound margins as analgesia. With a servo-controlled heating blanket, a body core temperature close to 37.0 °C was maintained throughout surgery. After a midline neck incision, standardized silicon rubber-coated 6.0 nylon monofilament (#6023910PK10; Doccol, Sharon, MA, USA) was inserted into the right common carotid artery and advanced via the internal carotid artery to occlude the origin of the MCA. After 60 min, mice were re-anesthetized and the filament removed to allow reperfusion. To reduce infarct variability all mice were operated by the same operator. Operation time did not exceed 10 min. The reperfusion time accounted for 23 h.

### Triphenyltetrazolium chloride (TTC) staining

Edema-corrected stroke volumes were assessed 24 h after tMCAO, based on 2,3,5-triphenyltetrazolium chloride (TTC) staining (#T8877, Merck). Animals were killed 24 h after tMCAO and brains were cut into three 2-mm-thick coronal sections. Slices were stained for 20 min at 37.0 °C with 2.0% TTC to visualize the infarctions. Edema-corrected infarct volumes were calculated by planimetry (ImageJ software, National Institutes of Health, Bethesda, MD, USA) [15].

### Assessment of functional outcome

The global neurological deficits were quantified by conducting the Neuroscore, composed of the sum of the inverted Bederson score and the grip test, 24 h after stroke induction [16, 17].

### Exclusion criteria

Mice were excluded from endpoint analyses due to: (1) death before the predefined experimental endpoint; (2) Bederson score = 0 (24 h after tMCAO); (3) weight loss > 20% (24 h after tMCAO).

### bEnd5 cell culture

The commercially available cell line bEnd5 has been purchased from Merck (*b.End5*, #96091930). It has been established from primary brain EC of BALB/c mice. Immortalization has been carried out by infection of primary cells with retrovirus coding for the Polyomavirus middle T-oncogene. bEnd5 were grown in Dulbecco’s Modified Eagle Medium (DMEM) (high glucose, 4.5 g/L), supplemented with 10% fetal calf serum (FCS) and 1% l-glutamine (200 mM), in a humidified (95%) 37.0 °C incubator with 5.0% CO_2_ and 21% O_2_. bEnd5 were plated in 75 cm^2^ culture flasks and subcultured using 0.25% (w/v) trypsin in 0.02% (w/v) EDTA at 80% confluence. Media was changed every 2 d and cells chosen for experimentation were passaged between 18 and 24 times. For Western Blot/zymography analysis bEnd5 were subcultured in 24-well plates, for microscopy in ibidi slides until confluency.

### Oxygen and glucose deprivation (OGD)

Confluent monolayers of bEnd5 were exposed to hypoxic (3.0% O_2_, 95% humidity, 5.0% CO_2_, 37.0 °C) and aglycemic conditions by replacing the culture medium with low glucose-medium (hypoxic DMEM low glucose with 1% l-glutamin without FCS). OGD medium was pre-incubated for 24 h under hypoxic conditions before administration.

### bEnd5 treatment

bEnd5 were treated with varying concentrations of the inflammasome-inhibitor MCC950. MCC950 was dissolved as described above. As vehicle treatment, the same amount of pure culture or OGD medium was added. The investigators were blinded to the group allocation.

### Western blot analysis

bEnd5 were cultured in 24-well plates until confluency. After cell lysis, the denatured protein was electrophoresed and transferred to a nitrocellulose membrane. Membranes were blocked for 1 h and incubated with the primary antibody at 4.0 °C overnight. Thereafter, the membranes were incubated with horseradish peroxidase-conjugated Immunoglobulin G antibody (#111-035-045, 1:10,000, Dianova, Hamburg, Germany) at room temperature for 1 h. Proteins were detected using ECLplus (#NEL104001EA, PerkinElmer, Waltham, MA, USA) and the ChemiDocTM Touch Imaging System (Bio-Rad, Hercules, CA, USA). To control protein loading, membranes were incubated with a β-actin monoclonal antibody (expected mass ~42 kDa). After intense washing, membranes were blocked for 1 h and incubated with the primary β-actin antibody (#A5441, 1:250,000, Merck) at 4.0 °C overnight. Thereafter, membranes were incubated with horseradish peroxidase-conjugated Immunoglbulin G antibody (#715-035-150, 1:10,000, Dianova) at room temperature for 1 h. Actin was detected as described above. Bands were quantified by densitometric analysis using Image Lab Analysis Software Version 5.2.1. (Bio-Rad, Hercules, CA, USA).

### Zymography

MMP9 in the in vivo and in vitro model was detected by zymography as reported before [18]. In brief, either ipsilesional cortical or basal ganglial protein probes of tMCAO-treated mice or bEnd5 supernatants after 0 h, 5 h, 10 h, 24 h of OGD or normoxia were collected and mixed with equal volumes of sample buffer (20% Glycin, 4.0% SDS, 2 mM EDTA, 0.01% Bromophenol Blue, 125 mM Tris–HCl pH 6.8). Next, samples were loaded on a 10% sodium dodecyl sulfate–polyacrylamide gel electrophoresis (0.1% gelatin). Then, gels were treated twice with 2.7% Triton X-100 solution for 2 × 30 min, washed in water, and incubated in developing buffer (50 mM Tris, 200 mM NaCl, 5 mM CaCl_2_, 0.02% Brij-35, 40 mM Tris–HCl pH 7.5) at 37.0 °C overnight. Gels were stained with 0.25% (w/v) Coomassie brilliant blue in 25% isopropanol/10% acetic acid for 30 min and bleached with 50% methanol and 10% acetic acid for 30 min until bands with diminished staining appeared. Gel images were captured with the ChemiDocTM Touch Imaging System and subsequently analyzed with ImageJ Analysis Software 1.52a.

### Cytometric bead array

Cytometric Bead Arrays for CXCL1, CCL2, CCL5, and CXCL10 were performed with bEnd5 supernatants using LEGENDplex^TM^ fluorescent bead immunoassays according to the manufacturer’s instructions. The readout was performed with the BD FACSlyric^TM^ (Becton Dickinson, Franklin Lakes, NJ, USA).

### Enzyme-linked immunosorbent assay

To determine IL1b and IL18 levels, commercially available ELISA kits were used (Mouse IL-1 beta ELISA Kit, #ab197742, Abcam, Cambridge, UK; IL-18 Mouse ELISA Kit, #BMS618-3, Thermo Fisher Scientific, Waltham, MA, USA). Cell lysates were prepared as described above. All reagents were provided with the kits and the ELISAs were performed according to the manufacturer’s manual.

### Immunohistochemistry

For histology brain tissue was cut in 2-mm-thick coronal sections, embedded in Tissue-Tek OCT compound, and frozen. Brain sections were cut on a cryostat into 10 μm thin slices and used for all analysis. For immunohistochemistry, slices were post-fixated with ice-cold 100% methanol. Immunohistochemistry was performed according to standard procedures [12]. Mouse brains were stained for DAPI and TUNEL and with anti-NLRP3 (Anti-NLRP3/NALP3, mAb (Cryo-2), #AG-20B-0014, 1:100, Adipogen Life Sciences, San Diego, CA, USA), anti-CD31 (Anti-CD31 antibody, #ab28364, 1:100, Abcam), anti-Albumin (Anti-Albumin antibody, #ab106582, 1:100, Abcam). Cell cultures were dyed with TUNEL, DAPI, Propidium Iodide (PI) and antibodies against NLRP3 (Anti-NLRP3/NALP3, mAb (Cryo-2), #AG-20B-0014, 1:100, Adipogen Life Sciences), Caspase 1 (uncleaved) (Caspase 1 Monoclonal Antibody (5B10), #14-9832-82, 1:100, Thermo Fisher Scientific), the p20 subunit of cleaved Caspase 1 (anti-Caspase-1 (p20) (mouse), #AG-20B-0042, 1:200, Adipogen Life Sciences) and Zonula Occludens 1 (ZO-1) (ZO-1 Polyclonal Antibody, #61-7300, 1:1000, Thermo Fisher Scientific). Secondary antibodies were used in a dilution of 1:100. For measurement of albumin intensity, images at the level of the basal ganglia (0.5 mm anterior from bregma) of five different animals per group were recorded with a microscope (Leica DMi8, DMC 2900/DFC 3000 G camera control, LAS X software, Leica, Wetzlar, Germany). Subsequently, after converting the images into 16-bit black/white files, the intensity of the albumin staining was compared between the ipsilesional and contralesional hemispheres with ImageJ Analysis Software 1.52a. bEnd5 cultivated on ibidi slides were visualized with transmitted light microscopy and apoptotic bEnd5 with fluorescence measurements after PI (1:200) staining with the above-mentioned microscope. The red fluorescent cells were counted. The fluorescence intensity of anti-GSDMD and anti-ZO-1 stainings were measured as described above.

### Statistical analysis

Results are presented as box plots indicating the median, the 25th percentile, the 75th percentile, minimum and maximum. Error bars show the standard error of the mean. For statistical analysis, the GraphPad Prism 8 software was used. Data were tested for Gaussian distribution with the D’Agostino-Pearson omnibus normality test and then analyzed by one-way analysis of variance with *post* *hoc* Tukey adjustment for *p* values. If comparing only two groups paired or unpaired—depending on the respective question—two-tailed *t* tests were applied. Probability values <0.05 were considered to indicate statistically significant results.

## Results

### NLRP3 inhibition leads to a higher endothelial survival after OGD

**Fig. 1:**
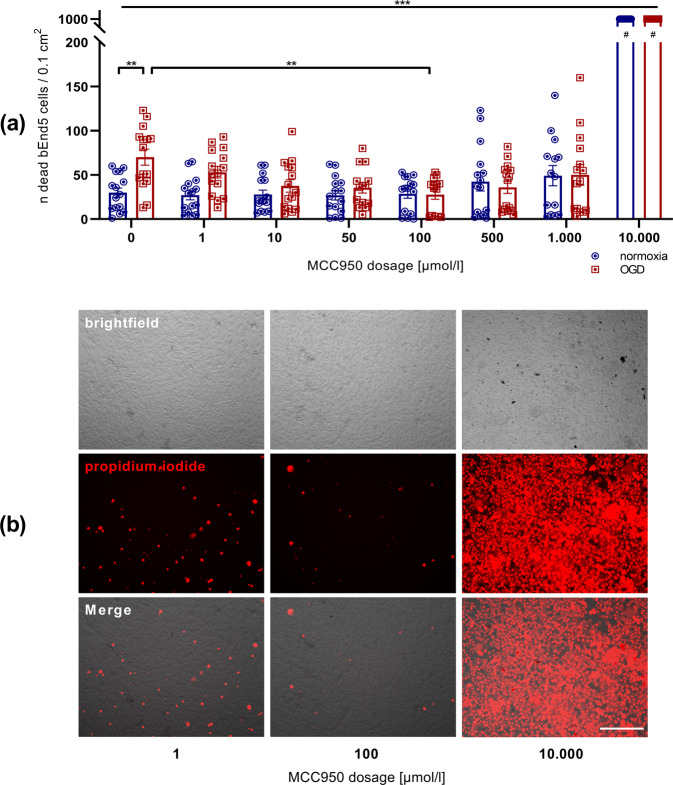
The comparison of bEnd5 after 24 h of either normoxia or OGD shows a significantly higher rate of cell death under OGD conditions. The MCC950 dilution series within the bEnd5 cell culture favors the 100 µmol/l MCC950 concentration with a significant reduction of cell death under OGD. **a** Number of apoptotic bEnd5 cells per 0.1 cm^2^ after 24 h of either normoxia (blue) or OGD (3.0% O_2_, 5.0% CO_2_, 95% humidity, 37.0 °C, 1 g/l glucose, red) depending on the concentration of the initial MCC950 treatment in comparison to untreated control cells (*n* = 15 out of three independent experiments). #: counting stopped at 1000 dead cells/0.1 cm^2^. **b** Representative microscopic brightfield images of bEnd5 after 24 h OGD and immunofluorescent staining of the propidium iodide (red) uptake of these bEnd5 cells as cell death marker in selected MCC950 concentrations. In all, ×20 objective; scale bar = 25 µm. Data were analyzed by one-way ANOVA with Tukey *post* *hoc* test. **p < 0.01; ***p < 0.001.

**Fig. 2:**
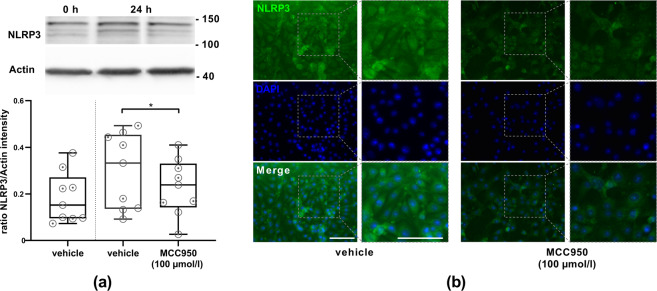
bEnd5 express NLRP3 protein. The amount of NLRP3 protein is dependent upon OGD as well as MCC950 (100 µmol/l) treatment. **a** Top: representative Western Blot depicting the amount of expressed NLRP3 (120 kDa) in bEnd5 lysates. To control protein loading β-actin was used (expected mass ~42 kDa). Bottom: ratio of NLRP3 protein band intensity to actin as loading control after 0 h and 24 h of OGD (3.0% O_2_, 5.0% CO_2_, 95% humidity, 37.0 °C, 1 g/l glucose) with vehicle or MCC950 (100 µmol/l) treatment (*n* = 9 out of three independent experiments). **b** Representative NLRP3 (green) and DAPI (blue) immunofluorescence stainings of bEnd5 after 24 h of OGD either with vehicle or with MCC950 (100 µmol/l) treatment. Adjacent ×2 magnification. In all, ×20 objective; scale bar = 25 µm. Data were analyzed by unpaired *t* test. *p < 0.05.

Our aim was to characterize the influence of the NLRP3-specific inflammasome-inhibitor MCC950 on EC. Therefore, we took advantage of the bEnd5 cell model that has frequently been shown to be suitable to study the responsiveness of brain EC under normoxic and ischemic conditions [19–21]. To visualize cell death PI was administered. At first, a significant increase of dead bEnd5 after 24 h of OGD in comparison to bEnd5 under normoxic conditions was observable. Subsequently, in order to check the effect of MCC950 on these bEnd5 under OGD, a dilution series of the NLRP3 inhibitor was accomplished. It could be clearly shown that a MCC950 concentration of 100 µmol/l leads to the highest bEnd5 survival rate within the OGD setting while higher concentrations seemed to have a toxic and lower concentration an insufficient effect (Fig. 1, Figure S1). To verify the *de* *facto* NLRP3 expression on bEnd5, Western Blot analysis of bEnd5 lysates was performed, firstly after normoxic conditions and secondly after 24 h of OGD either with or without MCC950 treatment. An NLRP3 expression could be detected in general, with an NLRP3 protein upregulation after OGD. Under OGD conditions, significantly fewer NLRP3 was measurable after MCC950 application in comparison to vehicle treatment. NLRP3 immunofluorescence stainings of bEnd5 cell cultures after 24 h OGD revealed a reduced NLRP3 expression after MCC950 treatment, too (Fig. 2).

### NLRP3 mediates pyroptosis of EC

**Fig. 3:**
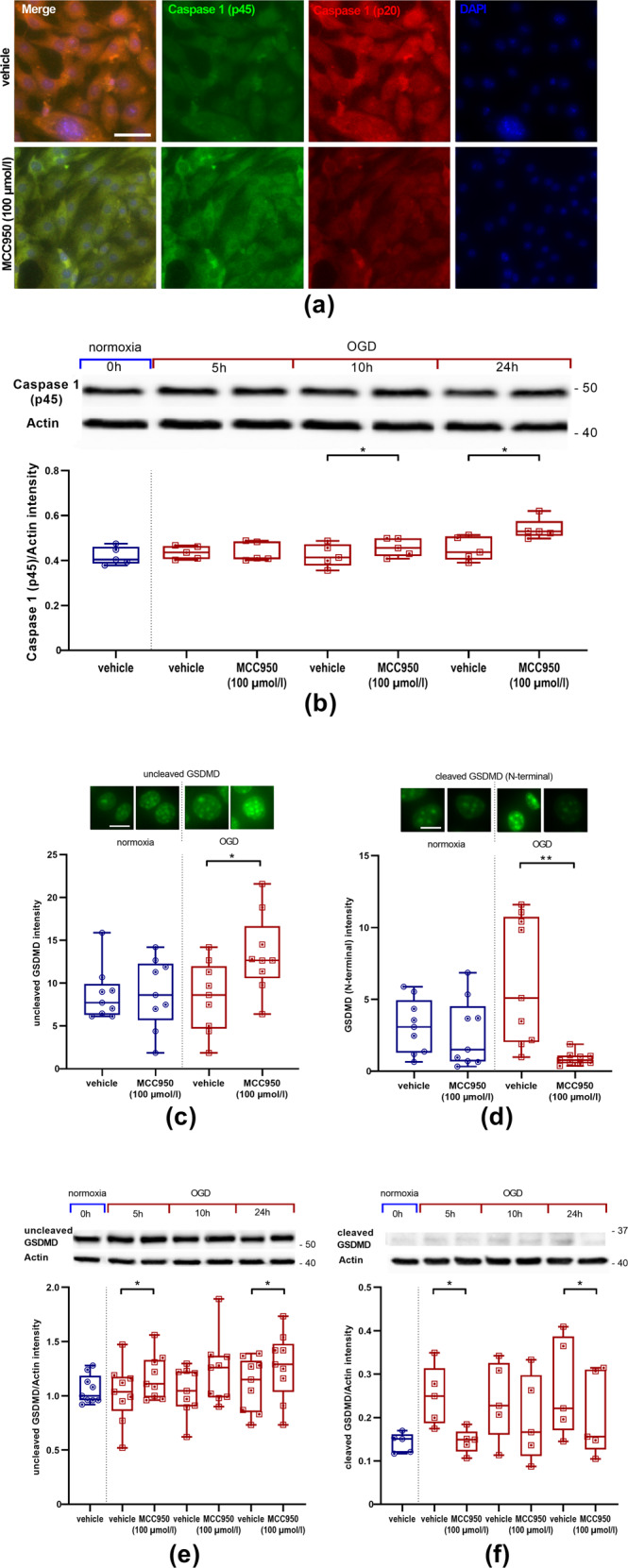
MCC950 (100 µmol/l) treatment reduces pyroptosis, indicated by lower levels of active Caspase 1 (p20), higher levels of pro-Caspase 1, elevated uncleaved GSDMD as well as reduced cytotoxic N-terminal GSDMD. **a** Representative pro-Caspase 1 (p45) (green), active Caspase 1 (p20) (red), and DAPI (blue) immunofluorescence stainings of bEnd5 after 24 h OGD (3.0% O_2_, 5.0% CO_2_, 95% humidity, 37.0 °C, 1 g/l glucose, red) either with vehicle or with MCC950 treatment. In all, ×20 objective; scale bar = 25 µm. **b** Top: representative Western Blot depicting the amount of expressed pro-Caspase 1 (45 kDa) in bEnd5 lysates. To control protein loading β-actin was used (expected mass ~42 kDa). Bottom: ratio of pro-Caspase 1 (p45) protein band intensity to actin as loading control after 0 h, 5 h, 10 h, and 24 h of OGD with vehicle or MCC950 (100 µmol/l) treatment (*n* = 5 out of three independent experiments). **c**, **d** Top: representative immunofluorescence stainings of either uncleaved GSDMD or GSDMD (N-terminal) (green). Bottom: either uncleaved GSDMD or GSDMD (N-terminal) intensity of bEnd5 cell cultures after 24 h OGD with either vehicle or MCC950 (100 µmol/l) treatment (*n* = 9 out of four independent experiments). In all, ×40 objective; scale bar = 5 µm. **e**, **f** Top: representative Western Blot depicting the amount of expressed uncleaved (52 kDa) or cleaved (N-terminal) (32 kDa) GSDMD in bEnd5 lysates. To control protein loading β-actin was used (expected mass ~42 kDa). Bottom: ratio of either uncleaved GSDMD or cleaved (N-terminal) GSDMD protein band intensity to actin as loading control after 0 h, 5 h, 10 h, and 24 h of OGD with vehicle or MCC950 (100 µmol/l) treatment (*n* = 9 or five out of three independent experiments). Data were analyzed by paired *t* test. *p < 0.05; **p < 0.01.

To further specify the type of cell death accompanying the upregulated NLRP3 expression in EC under OGD conditions, we explored evidence for pyroptosis. As a first finding, we demonstrated immunohistochemically elevated levels of the activated form (p20) and reduced levels of the pro-form (p45) of Caspase 1 in vehicle-treated cells after OGD (Fig. 3a, b). Moreover, MCC950 (100 µmol/l) treatment led to increased immunohistochemical intensities in the staining of uncleaved GSDMD and reduced intensities of the cytotoxic cleaved N-terminal GSDMD (Fig. 3c, d). These protective effects of MCC950 were confirmed in the respective Western Blot analyses (Fig. 3e, f) [22–24]. With that, we could clarify that the increased levels of uncleaved GSDMD are a reliable indirect indicator for reduced pyroptosis after MCC950 therapy.

### NLRP3 inhibition attenuates post-ischemic chemokine, cytokine, and MMP9 release

**Fig. 4:**
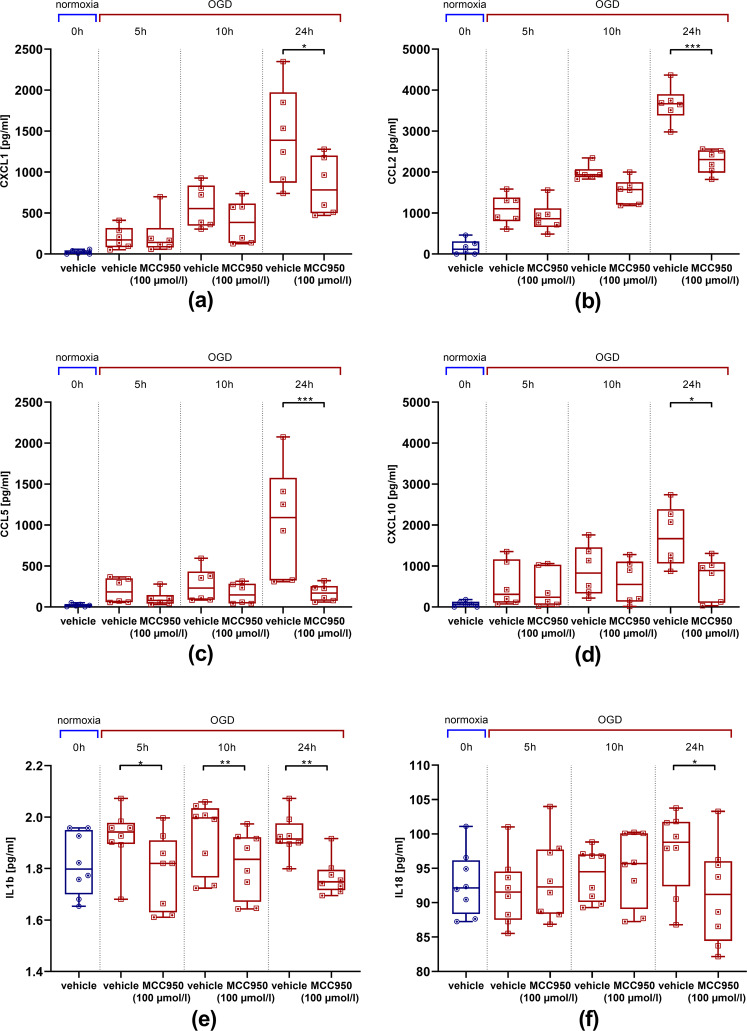
bEnd5 express less pro-inflammatory chemokines and cytokines after MCC950 (100 µmol/l) treatment. **a**–**d** Cytometric bead array to detect endothelial chemokine release depending on OGD (3.0% O_2_, 5.0% CO_2_, 95% humidity, 37.0 °C, 1 g/l glucose, red) duration and treatment regime. **a** CXCL1 protein concentration (*n* = 6 out of three independent experiments), **b** CCL2 (MCP-1) protein concentration (*n* = 6 out of three independent experiments), **c** CCL5 (RANTES) protein concentration (*n* = 6 out of three independent experiments) and **d** CXCL10 (IP10) protein concentration (*n* = 6 out of three independent experiments) at baseline under normoxic conditions as well as after 5 h, 10 h, and 24 h of OGD with vehicle or MCC950 treatment. **e**–**f** ELISA to measure endothelial cytokine release depending on OGD duration and treatment regime. **e** IL1b protein concentration (*n* = 8 out of four independent experiments), **f** IL18 protein concentration (*n* = 8 out of four independent experiments). Data were analyzed by unpaired *t* test. *p < 0.05; **p < 0.01; ***p < 0.001.

We aimed to evaluate the impact of the NLRP3 inhibition on the release of pro-inflammatory chemokines by EC under ischemic pressure. Therefore, a cytometric bead array was performed to measure the protein concentrations of the endothelial pro-inflammatory chemokines CXCL1, CCL2 (MCP-1), CCL5 (RANTES), and CXCL10 (IP10) depending on the duration of OGD and treatment regime. The expression of the respective proteins was matched between an initial value at 0 h under normoxic conditions and MCC950/vehicle-treated bEnd5 after 5 h, 10 h, and 24 h of OGD. Upregulation of pro-inflammatory chemokines could be detected in vehicle-treated bEnd5 that reached statistical significance after 24 h of OGD (Fig. 4a–d). The same was evaluated with regard to pro-inflammatory cytokines related to the pyroptosis pathway: again a significant reduction of IL1b protein concentration already after 5 h of OGD and of IL18 after 24 h of OGD could be demonstrated after MCC950 (100 µmol/l) treatment compared with vehicle treatment (Fig. 4e–f).

**Fig. 5:**
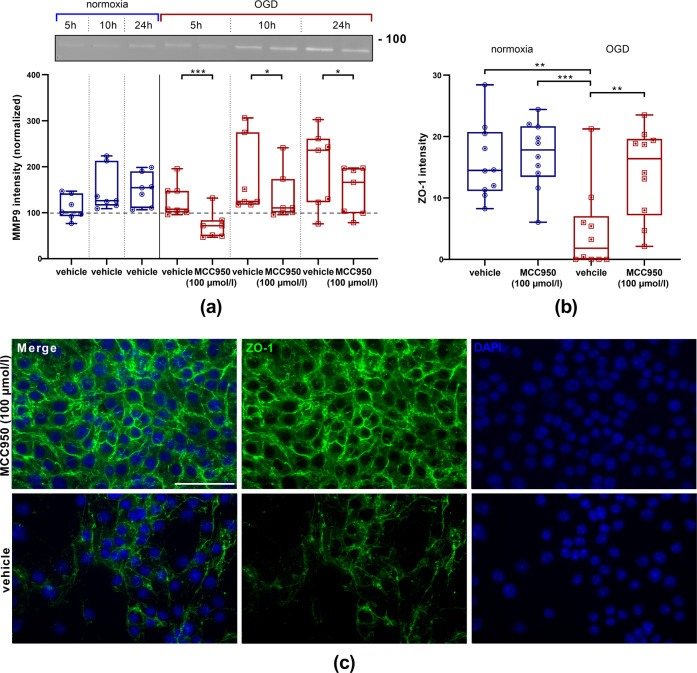
MMP9 reduction and ZO-1 enhancement after MCC950 (100 µmol/l) treatment. **a** Top: representative zymography with Coomassie staining depicting the amounts of secreted MMP9 (92 kDa) in bEnd5 supernatants after 5 h, 10 h, or 24 h of normoxia (blue) or OGD (3.0% O_2_, 5.0% CO_2_, 95% humidity, 37.0 °C, 1 g/l glucose, red) either with vehicle or MCC950 (100 µmol/l) treatment. Bottom: the intensity of MMP9 within the bEnd5 supernatants were normalized to the intensity of the probes at 0 h under normoxic conditions (dashed line, *n* = 7 out of three independent experiments). Data were analyzed by unpaired *t* test. *p < 0.05. ***p < 0.001. **b** Intensities of ZO-1 immunohistochemical stainings of bEnd5 under normoxia or OGD with either vehicle or MCC950 (100 µmol/l) treatment. Data were analyzed by one-way ANOVA with Tukey *post* *hoc* test. **p < 0.01; ***p < 0.001. **c** Representative ZO-1 (green) and DAPI (blue) immunofluorescence stainings of bEnd5 after 24 h of OGD either with vehicle or with MCC950 (100 µmol/l) treatment. ×20 objective; scale bar = 50 µm.

Moreover, zymography was performed with bEnd5 supernatants. Monocultured EC displayed a time-dependent increase of MMP9 levels after OGD [18, 25]. Within the MCC950-treated groups a significant decrease in comparison with vehicle-treated cells was observable (Fig. 5a). Furthermore, ZO-1 was stained and showed a significantly higher immunofluorescent intensity after MCC950 treatment, demonstrating that the effects of NLRP3 inhibition are not limited to higher survival of EC but also involve tight junction proteins (Fig. 5b, c).

### NLRP3 inhibition mitigates post-ischemic BBB disruption and cell death within the vascular compartment in vivo

**Fig. 6:**
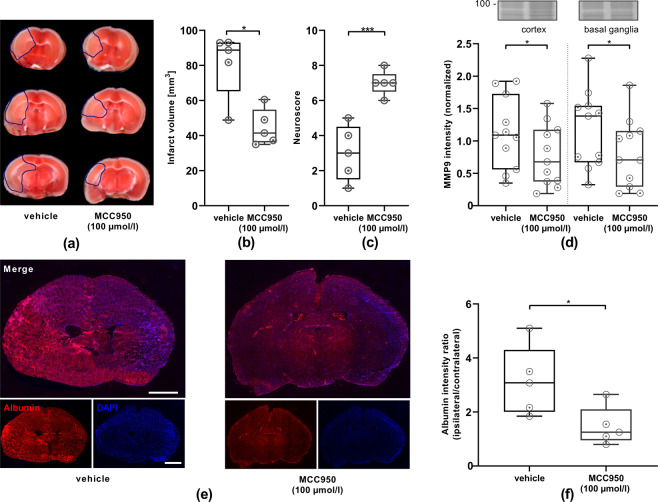
Treatment with the inflammasome-inhibitor MCC950 (100 µmol/l) reduces stroke severity, improves neurological outcome, reduces MMP9 levels, and lessens BBB disruption. **a** Representative 2,3,5-triphenyltetrazolium chloride staining of three consecutive coronal brain sections of vehicle- and MCC950 (100 µmol/l) treated mice euthanized 24 h after tMCAO. The infarcts (circled by blue line) are smaller in the MCC950 (100 µmol/l)-treated mice, which could be confirmed by **b** infarct volumetry (*n* = 5). **c** Neurologic scores were performed 1 d after tMCAO (*n* = 5). **d** Top: representative zymography with Coomassie staining depicting the amounts of secreted MMP9 (92 kDa) in ipsilesional cortical or basal ganglial brain lysates 1 d after tMCAO with and without NLRP3 inhibition (MCC950 100 µmol/l). Bottom: densitometric quantification of MMP9 within the brain lysates with normalization to the intensity of the total protein of the respective probe as displayed by the Coomassie staining (*n* = 11). **e** Representative immunohistological staining of albumin (red) and nuclei (DAPI, blue) of coronal brain slices 1 d after tMCAO in a vehicle (left) and MCC950 (100 µmol/l) treated mouse (right) using ×5 objective. scale bar = 2 mm. **f** Ratio of ipsilesional to contralesional albumin intensity of coronary brain slices 1 d after tMCAO in vehicle and MCC950 (100 µmol/l) treated mice (*n* = 5). Data were analyzed by unpaired *t* test. *p < 0.05; ***p < 0.001.

**Fig. 7:**
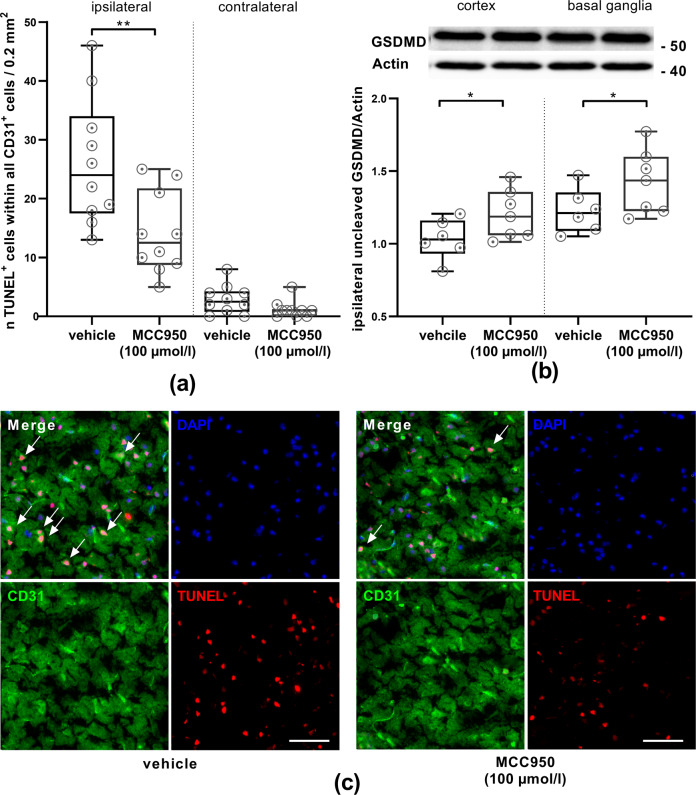
MCC950 (100 µmol/l) treatment reduces cell death within the vascular compartment after IS. Uncleaved GSDMD levels indicate reduced pyroptosis after inflammasome inhibition. **a** The number of CD31- and TUNEL-positive cells within all CD31-positive cells (ipsilateral or contralateral) of vehicle or MCC950 (100 µmol/l) treated animals on day 1 after tMCAO (*n* = 10, two per animal). **b** Uncleaved GSDMD (52 kDa) protein content in the cortex or basal ganglia of vehicle or MCC950 (100 µmol/l) treated mice. For densitometric quantification actin was used as a loading control (expected mass ~42 kDa) (*n* = 7). **c** Representative brain sections from vehicle (left) or MCC950 (100 µmol/l) (right) treated mice 24 h after tMCAO immunolabeled for the marker CD31 (green), TUNEL (red) to depict apoptosis and DAPI (blue) for nuclei. White arrows highlight triple-positive cells. In all, ×20 objective, scale bar = 100 µm. Data were analyzed by unpaired *t* test. *p < 0.05; **p < 0.01.

To translate these findings in an in vivo model, the tMCAO model was conducted with C57Bl/6N WT mice either with or without MCC950 treatment. Stroke volumes were diminished by ~40% and functional outcome improved significantly as revealed by the neuroscore on day 1, compared with vehicle-treated control animals (Fig. 6a–c). A key parameter characterizing neuroinflammatory processes within IS is the breakdown of the BBB measured by intracerebral albumin extravasation [26, 27]. Comparing the immunohistochemical albumin intensity 24 h after tMCAO a significant 50% reduction of the ipsilateral to contralateral albumin intensity ratio could be detected after MCC950 treatment (Fig. 6e–f). Moreover, zymography was performed to analyze MMP9 levels after IS [18, 28]. A significant reduction of MMP9 release in the ipsilateral cortices and basal ganglia of MCC950-treated mice compared with vehicle treatment was observable, comparable to the in vitro results (Fig. 6d). At the same time, we were able to depict a significant reduction of cell death within CD31-positive EC in the ischemic brain after MCC950 treatment. This reduction was accompanied by an increase of inactive, uncleaved GSDMD as shown in the cell culture model before (Fig. 7).

## Discussion

As a principal finding, we show that inhibition of the NLRP3 inflammasome with MCC950 reduces EC death—and here in particular pyroptosis—during IS. Within the last years, the NLRP3 inflammasome-mediated pyroptosis has been identified as a potential cause of EC death [10]. So far, a direct contribution of NLRP3-mediated inflammatory processes in EC had mainly been described in the context of systemic vascular endothelial dysfunction [10, 28]. In 2015, the NLRP3 inflammasome-inhibitor MCC950 that specifically inhibits the NLRP3 activation and IL1b, IL18, and GSDMD secretion by preventing the NLRP3-induced ASC oligomerization was firstly described [29]. The studies were performed in macrophages. Later, we were able to highlight the efficacy of MCC950 also in neurons [9]. Recently, the beneficial effect of MCC950 on the BBB integrity after intracerebral hemorrhage has been demonstrated [30]. But the potential impact of NLRP3 inflammasome activation in highly specialized brain EC on IS-induced endothelial dysfunction and subsequent BBB breakdown had remained elusive.

In previous as well as in the present study, we have shown the potential of global NLRP3 inhibition to stabilize the BBB in an in vivo stroke model [9]. We now extend these findings by specifically addressing the role of endothelial NLRP3 under hypoxia/hypoxemia. We took advantage of bEnd5 cells, which are highly sensitive to short exposures to OGD, to study the consequences of NLRP3 activation in EC without confounding hemodynamic variables and cell types [20, 31]. After determining the optimal concentration of MCC950 treatment within the OGD setting, we could show that bEnd5 express NLRP3 protein, and importantly could be downregulated after MCC950 treatment during OGD. This was associated with higher survival of the EC as well as a stabilization of surrounding tight junctions. Cell death relating to inflammasome activation is attributed to pyroptosis [32]. We found upregulated cleaved Caspase 1 as well as respective GSDMD levels—the latter both in vitro and in vivo. In addition, we could show that NLRP3 inhibition reduced the secretion of pro-inflammatory chemokines and cytokines in vitro. This is in good agreement with previous in vivo findings depicting decreased immune cell infiltration into the ischemic hemisphere after tMCAO and MCC950 treatment [9]. Besides chemokines and cytokines, MMPs are a driving force for BBB destructing processes, too. A significant increase in MMP9 expression after rt-PA administration, accompanied by BBB disruption and ultimately hemorrhagic transformation, had been described [33]. In clinical studies, MMP9 has been shown to correlate with an increased risk of mortality and major disability as well as infarct volume growth after IS [34]. In our study, treatment of bEnd5 with MCC950 decreased the amount of MMP9 secretion during OGD. To close the loop, we analyzed MMP9 expression in our tMCAO mice and could present the very same outcome: NLRP3 inhibition reduced the amount of MMP9 in the ischemic hemispheres.

The positive effect of NLRP3 inhibition on the BBB integrity after IS can thus be explained by a dual mechanism: [1] Protection of the BBB itself due to fewer pyroptosis of EC and [2] less pro-inflammatory signaling cascades leading to fewer local inflammation. Which target actually produces therapeutic effects is a recurrent question in the NLRP3 inflammasome research [35]. With our results, we can clearly outline the detrimental role of endothelial NLRP3 in IS.

Besides its role in the acute inflammation in IS, the NLRP3 inflammasome has been demonstrated to contribute to chronic inflammatory processes resulting in premature endothelial senescence [36, 37]. Interestingly, the incidence of Alzheimer’s disease (AD) increases several-fold after acute IS [38, 39]. This might be explained at least to some extent by the contribution of inflammatory mediators to secondary brain damage in both AD and stroke [40]. The NLRP3 inflammasome is able to sense the accumulation of danger-associated molecular patterns during aging and mediates pro-inflammatory cascades. The inhibition of abnormal NLRP3-activity during aging has been shown to attenuate age-associated innate immune activation, age-related chronic diseases, and prolong healthspan [37]. It is therefore promising that the acute treatment with NLRP3 inhibitors such as MCC950 does not only lessen the neuroinflammation in IS and improve the post-stroke neurologic outcome but also reduce the incidence of AD after IS.

In conclusion, our findings establish the NLRP3 inflammasome as a major mediator of inflammation and pyroptosis in brain EC that ultimately plays a key role in BBB breakdown in IS. Therefore, NLRP3 represents a potential pharmacological target in acute IS therapy.

## Supplementary information

Supplementary Information

## Data Availability

Any additional data will be provided upon reasonable request.
