# Supplementary material for: NLPR3 inflammasome inhibition alleviates hypoxic endothelial cell death in vitro and protects blood–brain barrier integrity in murine stroke
Source: Cell Death Dis. 2021 Dec 20;13(1):20. doi: 10.1038/s41419-021-04379-z (PMC8688414; doi:10.1038/s41419-021-04379-z)
Supplement: Supplementary file 1 — Supplementary Information [file 41419_2021_4379_MOESM1_ESM.pdf]

Supplementary Information

bEnd5 survival during oxygen and glucose deprivation.

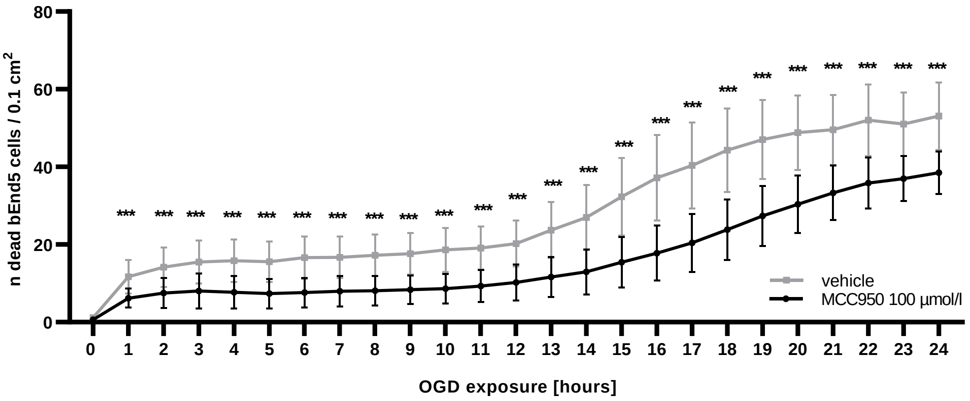

Figure S1: Survival of bEnd5 throughout 24 h of OGD treated with either vehicle or MCC950 (100 µmol/l). The survival after MCC950 treatment is significantly higher. *The number of apoptotic bEnd5 cells per 0.1 cm² hourly until 24 h of OGD (3.0% O_2, 5.0% CO_2, 95% humidity, 37.0°C, 1 g/l glucose) depending on the treatment regime: MCC950 (100 µmol/l) treatment (black) in comparison to vehicle treated control cells (grey) (n = 10 out of 3 independent experiments). Data was analyzed by 1-way ANOVA with Tukey post hoc test. **p < 0.01; ***p < 0.001.*

Materials

10 mg of the inflammasome-inhibitor MCC950 (NLRP3 Inhibitor, MCC950, #538120, Merck, Darmstadt, Germany) were dissolved in 1 ml 1 x phosphate buffered saline (PBS) (Dulbecco′s Phosphate Buffered Saline, #D8537, Merck). In the murine model, MCC950 was diluted with 1 x PBS to the concentration of 50 mg/kg body weight. For the *in-vitro* model MCC950 was further diluted with cell culture medium to 1 µmol/l, 10 µmol/l, 50 µmol/l, 100 µmol/l, 500 µmol/l, 1.000 µmol/l and 10.000 µmol/l. Dulbecco’s Modified Eagle’s Medium (DMEM) high glucose (4,5 g/l) (Dulbecco′s Modified Eagle′s Medium - high glucose, #D5671), DMEM low glucose (1,0 g/l) (Dulbecco′s Modified Eagle′s Medium - low glucose, #D5921), sterile water (Water, #W3500), L-Glutamine (L-Glutamine Solution 200 mM, #59202C), Trypsin (Trypsin-EDTA solution 0,25%, #T4049), anti-β-Actin (Monoclonal Anti-ß-Actin antibody produced in mice – clone AC-15, ascited fluid, #A5441), Propidium Iodide (PI) (Propidium iodide solution – solution 1.0 mg/ml in water, #P4864) and the TUNEL Kit (*In Situ* Cell Death Detection Kit, TMR red, #12156792910) were all purchased by Merck. Bovine serum (Sterile Plasma Derived Bovine Serum, #60-00-850) was delivered by First Link (UK) Ltd. (Birmingham, UK). The T75-cell culture flasks (Cellstar Cell Culture Flasks, 75 cm², #658170) were provided by Greiner Bio-One GmbH (Frickenhausen,

Germany), the 6- (Nunc™ Cell-Culture, 6-Well, #150239) and 24-well plates (Nunc™ Cell-Culture, 6-Well, #144530) as well as anti-NLRP3 (NLRP3 Recombinant Rabbit Monoclonal Antibody (SC06-23), #MA5-32255), DAPI (ProLong™ Gold Antifade Mountant with DAPI, #P36931) and as secondary antibodies Alexa Fluor™ 488 goat anti-mouse IgG (Goat anti-Mouse IgG (H+L), Alexa Fluor 488, #A11001), 488 donkey anti-rabbit IgG (Donkey anti-Rabbit IgG (H+L), Alexa Fluor 488, #A21206), 647 goat anti-rat IgG (Goat anti-Rat IgG (H+L), Alexa Fluor 647, #A21247), 546 goat anti-rabbit IgG (Goat anti-Rabbit IgG (H+L), Alexa Fluor 546, #A11035), 488 donkey anti-rat IgG (Donkey anti-Rat IgG (H+L), Alexa Fluor 488, #A21208) and the ELISA kits for IL18 (IL-18 Mouse ELISA Kit, #BMS618-3) were provided by Thermo Fisher Scientific (Waltham, MA, USA). 6-channel slides (µ-Slide VI 0.4, #80606) coated with ibidiTreat® were purchased by ibidi (Gräfelfing, Germany). Anti-GSDMD (uncleaved) (Recombinant Anti-GSDMD antibody, #ab219800) and anti-GSDMD (N-terminal) (Recombinant Anti-cleaved N-terminal GSDMD antibody , #ab215203) and the ELISA kit for IL1b (Mouse IL-1 beta ELISA Kit, #ab197742) were delivered by Abcam (Cambridge, UK). CXCL1, CCL2, CCL5 and CXCL10 were determined by a LEGENDplex™ fluorescent bead immunoassay (Mouse Anti-Virus Response Panel, #740622), purchased by BioLegend (San Diego, CA, USA).
